# A Multi-Scale Natural Scene Text Detection Method Based on Attention Feature Extraction and Cascade Feature Fusion

**DOI:** 10.3390/s24123758

**Published:** 2024-06-09

**Authors:** Nianfeng Li, Zhenyan Wang, Yongyuan Huang, Jia Tian, Xinyuan Li, Zhiguo Xiao

**Affiliations:** 1 College of Computer Science and Technology, Changchun University, No. 6543, Satellite Road, Changchun 130022, China; 2 School of Computer Science Technology, Beijing Institute of Technology, Beijing 100811, China

**Keywords:** text detection, attention mechanism, cascaded feature fusion, deep learning

## Abstract

Scene text detection is an important research field in computer vision, playing a crucial role in various application scenarios. However, existing scene text detection methods often fail to achieve satisfactory results when faced with text instances of different sizes, shapes, and complex backgrounds. To address the challenge of detecting diverse texts in natural scenes, this paper proposes a multi-scale natural scene text detection method based on attention feature extraction and cascaded feature fusion. This method combines global and local attention through an improved attention feature fusion module (DSAF) to capture text features of different scales, enhancing the network’s perception of text regions and improving its feature extraction capabilities. Simultaneously, an improved cascaded feature fusion module (PFFM) is used to fully integrate the extracted feature maps, expanding the receptive field of features and enriching the expressive ability of the feature maps. Finally, to address the cascaded feature maps, a lightweight subspace attention module (SAM) is introduced to partition the concatenated feature maps into several sub-space feature maps, facilitating spatial information interaction among features of different scales. In this paper, comparative experiments are conducted on the ICDAR2015, Total-Text, and MSRA-TD500 datasets, and comparisons are made with some existing scene text detection methods. The results show that the proposed method achieves good performance in terms of accuracy, recall, and F-score, thus verifying its effectiveness and practicality.

## 1. Introduction

As one of the main forms of information transmission, text holds an extremely important position in today’s society. Natural scene text detection is a crucial research topic in the field of deep learning, whose primary role is to accurately detect and locate the position of text in natural scenes, ensuring that users can promptly obtain reliable, authentic, and secure information. Accurate detection results play a crucial role in various research directions, including license plate recognition, text recognition, blind guidance, and document translation. In recent years, with the rapid development of deep learning technology, text detection techniques have achieved remarkable results. For instance, Zhu et al. [1] modeled text instances in the frequency domain and proposed a novel Fourier contour embedding method to represent text contours of arbitrary shapes as compact features. Dai et al. [2] presented an end-to-end trainable contour-based regression framework to detect text of arbitrary shapes in natural images. Although Convolutional Neural Networks (CNNs) are widely used in object detection [3,4], there are still some challenges in applying CNNs to scene text detection due to issues such as the varying sizes and orientations of text [5]. On the one hand, some existing methods have problems such as insufficient feature extraction ability and poor perception of text areas, which may lead to false detections or missed detections when these methods are used to detect certain texts. On the other hand, although existing feature pyramid networks can effectively fuse extracted features, directly adding high- and low-dimensional features for fusion can result in information loss, preventing the full integration of extracted features and thus affecting the effectiveness of text detection. 

To address the aforementioned issues, this paper improves the original network based on the DBNet [6] text detection algorithm, enhancing its detection performance. To tackle the problems of text scale variation and insufficient feature extraction capabilities, we propose an improved attention feature fusion module, DSAF (Depthwise Separable Attentional Fusion), which incorporates depthwise separable convolution and an attentional fusion mechanism, embedding them into the backbone network to capture features of text at different scales and enhance the feature extraction capability of the network. To address the issue of inadequate feature fusion, we introduce an enhanced cascade feature fusion module, PFFM (Pyramid Feature Fusion Module), which enables the full fusion of features at different scales and expands the receptive field of the network through pyramid pooling modules and cascaded feature fusion. Subsequently, the information interaction of feature maps at different scales is achieved through the SAM (Subspace Attention Module), which enhances the detection effect of diversified texts. We trained and tested our proposed method on the multi-directional text ICDAR2015 dataset [7], the multi-lingual text MSRA-TD500 dataset [8], and the curved-text Total-Text dataset [9], comparing it with some existing detection methods. Experimental results demonstrate that our proposed method is effective in addressing the aforementioned issues.

## 2. Related Work

The traditional text detection methods mainly include those based on text texture features, connected domain detection, and sliding window detection. These methods have achieved certain results in early text detection, but they usually require manually designing complex detection features, feature classifiers, and post-processing procedures, making the process quite cumbersome. Additionally, text detection in natural scenes often needs to face challenges such as irregular sizes, blurred backgrounds, occlusions, and variations in aspect ratios. These traditional text detection methods increasingly fall short of meeting the demands for high accuracy and efficiency when confronted with these challenges. In recent years, the rise of deep learning technology has propelled the development of text detection, and many researchers have begun introducing deep learning methods into the study of scene text detection, leading to the emergence of numerous scene text detection algorithms based on deep learning. Existing scene text detection algorithms based on deep learning are generally divided into two categories: scene text detection algorithms based on regression and scene text detection algorithms based on segmentation.

In scene text detection algorithms based on regression, the detection results are usually represented by rectangular boxes. The principle is to generate some candidate boxes first and then optimize these candidate boxes through regression algorithms to obtain more accurate text instances. When these algorithms were proposed, they drew heavily on the ideas of classic algorithms such as SSD [10], Faster R-CNN [11], and Mask R-CNN [12]. In 2016, influenced by Faster R-CNN, a text detection algorithm named CTPN [13] was proposed. This algorithm decomposes long texts into multiple small texts and then adjusts the text detection boundaries using Recurrent Neural Networks (RNN) to enhance detection results. In 2017, some scholars improved upon the SSD object detection method by splitting long text lines into small text segments with directions, designing a multi-angle text detection algorithm called SegLink [14], which further enriched the application scenarios of text detection. Also in 2017, a natural scene text detection method called TextBoxes [15] was proposed based on the SSD object detection algorithm. This algorithm modifies the scale of convolutional kernels for text detection, significantly improving the efficiency of text detection. In 2018, based on TextBoxes, Liao et al. further proposed TextBoxes++ [16]. This algorithm improves the network architecture, modifies the size of the convolutional kernels for inclined text, and adopts a fully convolutional structure, making it adaptable to different scales of input. In the same year, continuing the idea of the Region Proposal Network (RPN) for object detection, Ma et al. addressed the limitations of RPN in horizontal text detection and proposed Rotation Region Proposal Networks (RRPN) [17] for arbitrary-oriented scene text detection. This allows the candidate boxes to better adapt to text regions while ensuring the efficiency of text detection. Also in 2018, Jiang et al. proposed a method called CNN(R2CNN) [18] for detecting arbitrary-oriented text in natural scene images, further enhancing the accuracy and stability of text detection. Although these scene text detection algorithms based on regression have relatively fast detection speeds, their representation of text in the detection results is limited, and their generalization ability is weak. Most regression-based text detection algorithms have poor detection results for text with arbitrary shapes in natural scenes, which greatly affects subsequent text recognition tasks.

Another type of scene text detection algorithm based on deep learning is the segmentation-based scene text detection algorithm. The principle of this algorithm is to divide each pixel in the image into text regions and non-text regions, detecting text information in natural scenes through pixel-level segmentation. Compared to scene text detection algorithms based on regression, this type of detection algorithm can represent the position of text with arbitrary shapes more accurately, thus becoming a commonly used text detection algorithm at present. In 2017, an efficient and accurate scene text detection algorithm called East [19] was proposed, which can perform fast and accurate text detection in natural scenes. Subsequently, in 2018, Wang et al. proposed the PSENet [20] algorithm, which generates the entire text bounding box by performing progressive scale expansion on the text kernel, further improving the accuracy of text detection. In the same year, a text detection algorithm called TextSnake [21], capable of detecting text with arbitrary shapes, was proposed. This algorithm achieves precise detection of scene text with arbitrary shapes by detecting the text centerline. Deng et al., inspired by SegLink, proposed a method for detecting scene text through sample segmentation [22]. In 2019, Wang et al. proposed the Pixel Aggregation Network (PAN) [23], which aims to improve network speed and achieve real-time detection through pixel-level aggregation operations, further enhancing the accuracy of text detection. Also in 2019, Xu et al. proposed the TextField [24] method by leveraging the distinct relative positions of adjacent pixels in different text instances, adopting a strategy to connect adjacent pixels to generate candidate text parts, thereby achieving rapid localization of text regions. Similarly, in 2019, the Mask-PAN [25] algorithm was proposed, which uses a pyramid attention network to enable the model to better focus on the contextual information of image text, further improving the performance of text detection. Baek et al. proposed the CRAFT [26] algorithm for text detection based on character probability prediction, effectively detecting text regions by detecting each character and the relationships between characters, enabling accurate detection of large-scale text instances with small receptive fields. Xie et al. proposed a method called SPCNET [27], which can effectively detect text with arbitrary shapes. Long et al. [28] proposed an end-to-end scene text detection method, which is more sensitive to character detection. In contrast, DBNet proposes a differentiable binarization method that applies adaptive binarization to each pixel, derives the binarization threshold from network learning, and trains the binarization process together with the segmentation network to significantly improve the efficiency of post-processing. By improving the DBNet model, this paper compensates for the shortcomings of the original network’s insufficient feature extraction ability and inadequate feature fusion, expands the receptive field of the network, and improves the text detection performance.

## 3. Method

**Figure 1:**
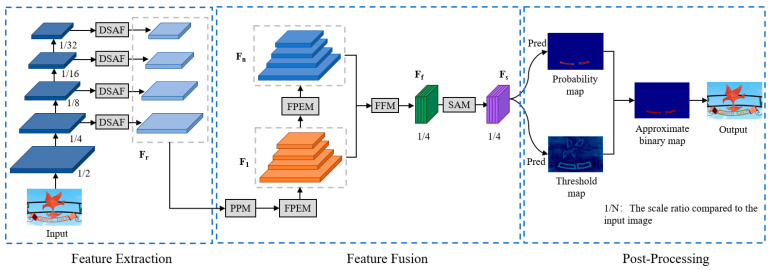
The overall framework of the proposed method.

The structure of our proposed model is illustrated in Figure 1, consisting of three main components: feature extraction, feature fusion, and post-processing. In the feature extraction component, we embed an improved attention-based feature fusion module (DSAF) into the feature extraction network. By combining global and local attention mechanisms, we aim to reduce the loss of feature information, enhance the network’s focus on text of different scales, and improve its feature extraction capabilities. In the feature fusion component, we utilize an improved cascaded feature fusion module (PFFM) to fully integrate the extracted features and expand the receptive field of the network. Subsequently, for the concatenated feature maps, we introduce the SAM [29] to establish cross-channel dependencies and achieve information interaction of multi-scale features in the spatial dimension. Finally, in the post-processing stage, we obtain an approximate binary map through differentiable binarization and generate detection results based on this map using a label generation algorithm.

### 3.1. Attention Feature Fusion

**Figure 2:**
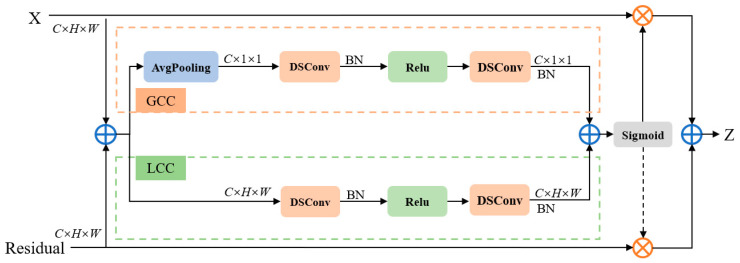
Details of the attention feature fusion module.

In the field of deep learning, the introduction of attention mechanisms is primarily aimed at simulating human perception mechanisms, enabling models to process input data more intelligently and flexibly, thereby improving their performance, generalization capabilities, and adaptability [30,31]. Attention mechanisms have become an indispensable tool for designing advanced deep-learning models across various tasks and domains. To preserve more text feature information during feature extraction, we propose an improved attention feature fusion module (DSAF) based on AFF [32] which uses depthwise separable convolution [33] and embeds it into the feature extraction network ResNet [34] to reduce the loss of feature information and increase the degree of attention to features of different scales. The structure of this module is illustrated in Figure 2.

Specifically, our improved attention feature fusion module combines global and local features to enhance the performance of the model. The global feature branch captures global features by adopting adaptive global average pooling and depthwise separable convolution and strengthens the focus on larger targets with a global distribution. On the other hand, local features capture specific details through depthwise separable convolution and highlight the characteristics of smaller targets [35]. Compared to SENet [36], which only employs a global attention mechanism, our module integrates text feature information of different scales on the channel dimension, thus mitigating the issues caused by text scale variations and enhancing the detection capability for small targets.

In this paper, we choose to fuse two input features, X and Residual. Among them, X represents the self-mapping and is a part of the input features, while Residual is the residual learned from the ResNet block and contains important information. The DSAF attention module first weights these two features to obtain X+Residual, and then sends the resulting feature to the GCC (Global Channel Context) module and the LCC (Local Channel Context) module for fusion, in order to comprehensively utilize both local and global information.

In the GCC module, for the input features, the global attention mechanism extracts the results of global features through depthwise separable convolutions, denoted as $Xg\in R^{C\times 1\times 1}$:

(1) Xg=DSConv(ReLU(BN(DSConv(Avg(X+Residual)))))

Avg stands for adaptive global average pooling, and DSConv represents depthwise separable convolution. Compared to ordinary convolution, depthwise separable convolution is able to capture text features of different scales while reducing the model size and computational cost. This improves adaptability to text regions with scale variations, making the model more robust and generalizable when dealing with diverse texts and complex scene texts. 

Similarly, in the LCC module, the local attention mechanism also extracts local feature results through depthwise separable convolutions, denoted as $Xl\in R^{C\times H\times W}$:

(2) Xl=DSConv(ReLU(BN(DSConv(X+Residual))))

The shape of Xl is identical to the input features, allowing it to maintain the spatial structure of the original features. This highlights the subtle features that carry significant meaning in the underlying representation, which is beneficial for enhancing the overall quality of feature expression.

GCC employs adaptive global average pooling, resulting in features with a height and width shape of 1×1. On the other hand, LCC maintains the same height and width dimensions as the original input features, namely H×W. Therefore, to fuse the global and local features, we adopt a broadcast addition operation to add the obtained global feature result Xg to the local feature result Xl, yielding a new feature $Xlg\in R^{C\times H\times W}$ that effectively integrates both global and local information. This is defined by the following formula:

(3) Xlg=Xg⊕Xl

The newly obtained feature Xlg is activated through the Sigmoid function and finally performs an element-wise multiplication operation with the smaller feature layer in the original input (here is X). This applies the global and local attention weights to the original input features, enabling the model to focus more on the information considered more important. The formula is as follows:

(4) Z=X⊗(Sigmoid(Xlg))

Here, we use the Sigmoid activation function to map each element’s value to the range of [0, 1], avoiding excessively large or small weights. The attention weights processed by the Sigmoid function are then used to perform a weighted fusion of the local and global features. This ensures that the fused features better reflect the information in the image, thereby improving the stability of the model.

### 3.2. Depthwise Separable Convolution

**Figure 3:**
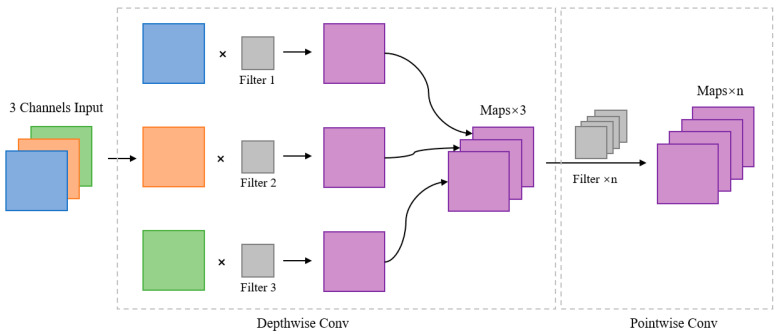
Details of depthwise separable convolution.

Depthwise separable convolution is divided into two parts: depthwise convolution and pointwise convolution. Depthwise convolution is used to extract spatial features, while pointwise convolution is used to extract channel features. The depthwise convolution in depthwise separable convolution can efficiently process each input channel. For a three-channel input, depthwise convolution splits the channels, convolves each channel with a separate convolution kernel, and obtains three feature maps through three convolution kernels. Then, the obtained three feature maps are subjected to pointwise convolution, using n 1 × 1 convolution kernels for dimensionality expansion to obtain n feature maps. For text detection, compared with pointwise convolution, depthwise separable convolution performs independent depthwise convolution on each channel and aggregates all channels through pointwise convolution before output. The structure of depthwise separable convolution is shown in Figure 3.

### 3.3. Pyramid Pooling Module (PPM)

**Figure 4:**
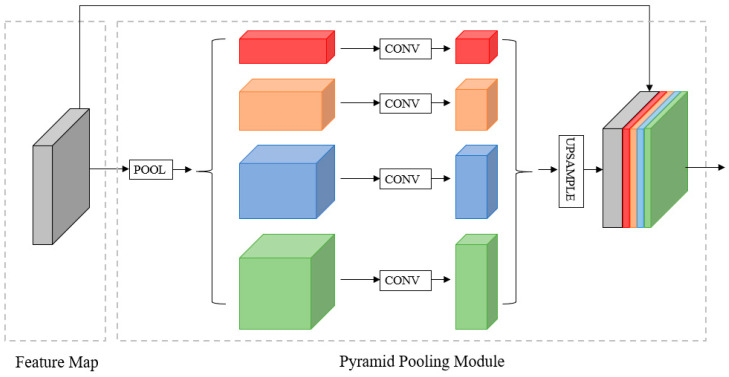
Details of the PPM.

To enhance the network’s detection capability for texts of different scales and enrich the ability of high-level features to represent text geometry, we incorporate a Pyramid Pooling Module (PPM) [37] after feature extraction, as illustrated in Figure 4. This module captures high-level feature information at various scales through pooling, resulting in multiple feature maps of different sizes. These feature maps are further convolved. Subsequently, the resulting feature maps are upsampled to obtain feature maps of the same size as before the pyramid module. Finally, these feature maps are concatenated in the channel dimension to form the final composite feature map. Therefore, the inclusion of the PPM module enables a more comprehensive utilization of multi-scale feature information, which is concatenated to fuse local and global high-level features, enriching the final feature representation. This module is part of PFFM.

### 3.4. Cascaded Feature Fusion

Text instances in natural scene images vary greatly, including text with different directions and sizes, which increases the difficulty of text detection. To fully fuse the extracted features and improve the robustness of the text detection network, we propose an improved Cascaded Feature Fusion Module (PFFM) to replace the feature fusion network in the original DBNet. This module effectively integrates shallow and deep features, further enhancing the robustness of the detector. PFFM consists of a pyramid feature enhancement module (PFEM) and a feature fusion module (FFM).

**Figure 5:**
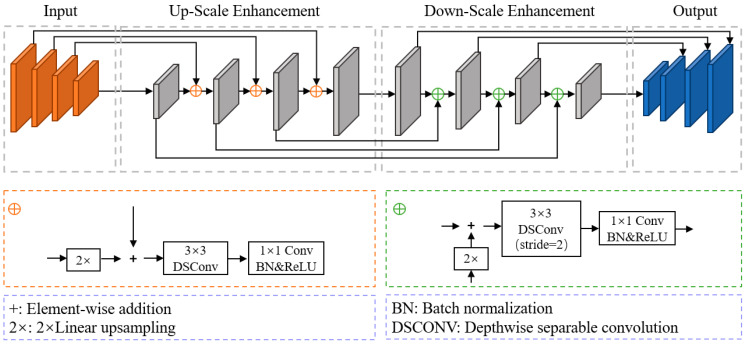
Details of the FPEM.

PFEM consists of PPM and FPEM. The FPEM exhibits a “U”-shaped structure with two components: up-scale enhancement and down-scale enhancement. The up-scale enhancement operates on the input feature pyramid, iteratively enhancing the feature maps to fully integrate shallow and deep features. The input for the down-scale enhancement is the feature pyramid generated by the up-scale enhancement. The down-scale enhancement generates output features through consecutive scaling and element-wise addition, and the resulting feature pyramid serves as the final output of the FPEM. The network structure of the FPEM is illustrated in Figure 5.

Similar to FPN, FPEM enhances features of different scales by fusing low-level and high-level information. However, unlike FPN, FPEM is a cascadable module. As the number of cascades increases, features of different scales are fused more thoroughly, and the receptive field of the features becomes larger. Additionally, compared to FPN, FPEM requires less computational overhead, making it more feasible for practical applications.

### 3.5. Feature Fusion Module (FFM)

**Figure 6:**
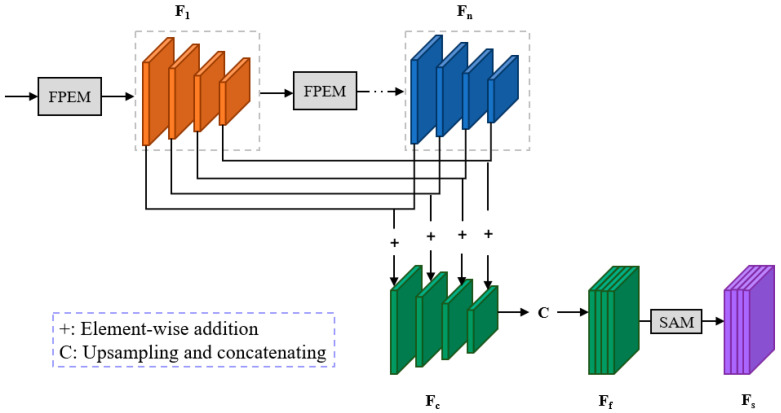
Details of the FFM.

The Feature Fusion Module is designed to integrate the feature maps $F_{1}$, $F_{2}$, and $F_{n}$ from different depths of the feature pyramid. As illustrated in Figure 4, we do not directly perform upsampling and concatenation; instead, we adopt an element-wise addition approach to combine the feature maps of corresponding scales. The last three feature maps in the merged feature pyramid $F_{c}$ undergo 2×, 4×, and 8× upsampling, respectively, to ensure they have the same resolution. Subsequently, all the feature maps in $F_{c}$ are concatenated to generate the feature map $F_{f}$. Finally, the final feature map $F_{s}$ is obtained through the SAM module. The network structure of the FFM is illustrated in Figure 6.

### 3.6. SAM

**Figure 7:**
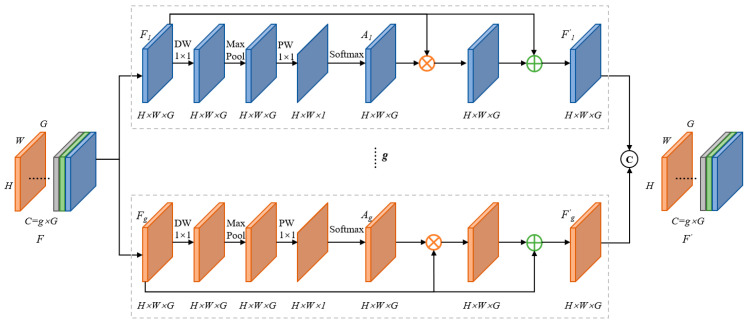
Details of SAM.

The text features differ greatly at different scales. To fully utilize features of varying sizes, this paper employs a SAM after feature fusion to achieve information interaction among multi-scale features in the spatial dimension. Its structure is illustrated in Figure 7.

Given an input feature map $F\in R^{C\times H\times W}$, the SAM initially divides the feature map into g groups, denoted as $\left[F_{1},F_{2},\cdots ,F_{\tilde{n}},\cdots ,F_{g}\right]$, where each group contains G channels of the feature map. $F_{\tilde{n}}$ represents the intermediate processing result of the feature map, and $A_{\tilde{n}}$ is defined as the attention feature map of $F_{\tilde{n}}$. The formula is described as follows:

(5) $$A_{\tilde{n}}=Softmax(PW(MaxPool(DW(F_{\tilde{n}}))))$$

In the above formula, MaxPool represents max pooling, DW stands for depthwise convolution with a kernel size of 1 × 1, and PW stands for pointwise convolution with a kernel size of 1 × 1. The attention feature map $A_{\tilde{n}}$ captures the non-linear dependencies between feature maps by learning cross-channel information. After the feature mapping process, each group of feature maps yields a set of feature maps $F_{\tilde{n}}$, and its formula is as follows:

(6) $${F^{\prime }}_{\tilde{n}}=(A_{\tilde{n}}⊗F_{\tilde{n}})⊕F_{\tilde{n}}$$

In the above formula, ⊗ represents element-wise multiplication, and ⊕ represents element-wise addition.

After obtaining the set of feature maps ${F^{\prime }}_{\tilde{n}}$, the final feature map is composed by concatenating each group of feature maps $\left[{F^{\prime }}_{1},{F^{\prime }}_{2},\cdots ,{F^{\prime }}_{\tilde{n}},\cdots ,{F^{\prime }}_{g}\right]$ along the channel dimension. The basic formula is as follows:

(7) $$F^{\prime }=concat([{F^{\prime }}_{1},{F^{\prime }}_{2},\cdots ,{F^{\prime }}_{\tilde{n}},\cdots ,{F^{\prime }}_{g}])$$

In the SAM, by dividing the channels into g sub-space feature maps, each sub-space feature map learns the high-frequency text features independently. This results in obtaining text feature weights from differently scaled feature maps, leading to the generation of a multi-scale fused feature map $F^{\prime }$. In this process, the sub-space feature maps with varying weights enable the feature map $F^{\prime }$ to contain more global text information, enabling the detection of text instances of different sizes and improving the accuracy of the network’s detection.

### 3.7. Differentiable Binary

After feature extraction and fusion, post-processing operations are required to transform the obtained features. The segmentation network generates a probability map, and, subsequently, this probability map needs to be converted into a binary map. In this paper, we employ a differentiable binarization approach to address this issue. The basic formula for differentiable binarization is as follows:

(8) $$B_{i,j}^{\prime }=\frac{1}{1+e^{-k(P_{i,j}-T_{i,j})}}$$

In the formula, (i, j) represents the coordinate point, $B^{\prime }$ stands for the approximate binary image, T is the adaptive threshold, and k serves as the scaling coefficient. During the training process, the value of k is primarily used to enhance the propagation gradient in backpropagation. In this paper, the value of k is set to 50.

### 3.8. Loss Function

In deep neural networks, the loss function serves a dual purpose: it measures the difference between the model’s predicted output and the true labels, and it provides feedback information necessary for the optimization algorithm. It is an indispensable component in the optimization process of deep learning models [38]. In this paper, the loss function we use is the $L_{1}$ loss function and the binary cross-entropy loss function [39]. The loss function in this paper is primarily composed of three parts: probability map loss $L_{s}$, threshold map loss $L_{t}$, and binarization map loss $L_{b}$. The basic formula is as follows:

(9) $$L_{1}=L_{s}+\alpha \times L_{b}+\beta \times L_{t}$$

In the above formula, weight parameter α is set to 1 and β is set to 10. The probability map loss $L_{s}$ and the binarization map loss $L_{b}$ adopt the binary cross-entropy loss function. The basic formula for this loss function is as follows:

(10) $$L_{s}=L_{b}=\sum_{i\in S_{l}}y_{i}\log x_{l}+(1-y_{l})\log(1-x_{l})$$

Meanwhile, we also adopt hard negative mining techniques [40] to address the imbalance between positive and negative samples. Here, $S_{l}$ indicates a ratio of 1:3 between positive and negative samples in the image. For the adaptive threshold map loss $L_{t}$, we employ the loss function $L_{1}$, which is formulated as follows:

(11) $$L_{t}=\sum_{t\in R_{d}}|y_{t}^{*}-x_{t}^{*}|$$

The above formula, $R_{d}$ is the index set of pixels inside the dilated polygon, and $y^{*}$ is the label of the threshold map.

## 4. Experiment and Analysis

### 4.1. Datasets

**Figure 8:**
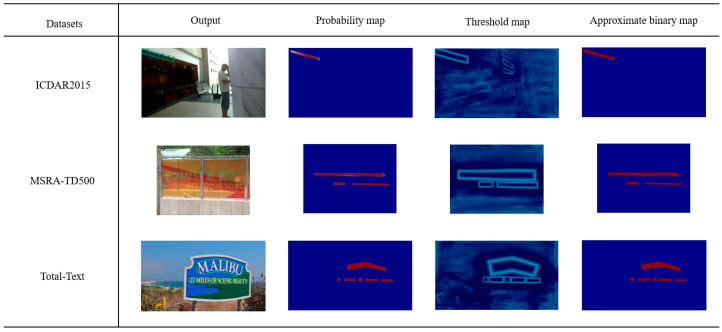
Visualization results on different types of datasets.

In this paper, we conducted training and testing on three widely used public datasets, including ICDAR2015 for multi-directional text, Total-Text for curved text, and MSRA-TD500 covering multi-lingual text. Figure 8 demonstrates the performance of our proposed algorithm on different types of text instances. The second column presents the probability maps, the third column displays the threshold maps, and the fourth column exhibits the binarized maps, jointly illustrating the processing procedures and outcomes of the algorithm.

The ICDAR 2015 dataset contains 1000 images for training and 500 images for testing. Primarily focusing on English text, it includes various scales and orientations of text images. This dataset is primarily composed of purely English text, including text images in different directions as well as some blurred images. Training and testing on this dataset enables the text detection algorithm to better adapt to a wide range of application scenarios.

The Total-Text dataset comprises 1255 images for training and 300 images for testing. It covers images from various scenes and environments, including streetscapes, billboards, signs, newspapers, and more. The text in this dataset exhibits diverse forms, with a majority of images featuring curved text. Training and testing on this dataset significantly improves the text detection algorithm’s perception of various shapes of text, enabling it to better adapt to different application scenarios.

The MSRA-TD500 dataset contains both Chinese and English text images, with 300 images for training and 200 images for testing. It covers both indoor and outdoor scenes. Indoor images mainly include signs and door numbers, while outdoor images involve scenes with complex backgrounds such as guideboards, billboards, and warning signs. Training and testing on this dataset effectively enhances the generalization ability of the text detection algorithm for different languages, enabling it to accurately detect and recognize text in various environments.

### 4.2. Experimental Configuration

In this study, we selected Python 3.7 as the programming language and used the deep learning framework Pytorch 1.5 to conduct experiments. The entire experimental process was accelerated with the NVIDIA GeForce RTX 3090 graphics card. Initially, we trained the network using the SynthText synthetic dataset [41] for 100 k iterations and then fine-tuned the model for 1200 epochs on real datasets based on the pre-trained model. We strictly adhere to the officially provided dataset without any modification, ensuring the accuracy of our experiments. In our experimental settings, we set the initial learning rate $l_{0}$ to 0.007, p to 0.9, weight decay to 0.0001, the number of cascaded fpems to 2, momentum to 0.9, and batch size to 16. Adam [42] was adopted as our training optimizer. The learning rate $l_{r}$ was continuously reduced using the following formula:

(12) $$l_{r}=l_{0}*{\left(1-\frac{epoch}{max\_epoch}\right)}^{p}$$

During this experiment, we employed three data augmentation techniques to expand the dataset, including random rotation, random splitting, and random flipping. Given the diversity of the dataset, small text regions were challenging to detect. Therefore, we ignored some excessively small text regions during the label creation process, excluding them from the training process. Since different scales of test images significantly impact detection performance, we maintained the aspect ratio of the test images during the inference stage and adjusted the size of the input images by setting an appropriate height for each dataset.

### 4.3. Evaluation Index

In the experiment, we determined the correctness of predictions by comparing whether the Intersection over Union (IOU) value between the predicted text box and the corresponding label box was greater than 0.5. The calculation formula is as follows:

(13) $$IOU=\frac{pred\cap gt}{pred\cup gt}$$

In the above formula, pred represents the area of the predicted text box, and gt represents the area of the label text box.

We use three main performance parameters, precision, recall, and F-score, to evaluate the detection performance of the model. The number of true text boxes predicted as text boxes is recorded as TP, the number of true text boxes predicted as background areas is recorded as FP, and the number of false text boxes predicted as background areas is recorded as FN. The comprehensive values for precision, recall, and F-score are calculated as follows:

(14) $$P=\frac{TP}{TP+FP}$$

(15) $$R=\frac{TP}{TP+FN}$$

(16) $$F=\frac{2\times P\times R}{P+R}$$

### 4.4. Ablation Experiment

To comprehensively evaluate the effectiveness of each module proposed in this paper and their impact on overall performance, we conducted ablation experiments on the improved model. The core idea of ablation experiments is to gradually add or modify specific parts of the model and observe the impact of these changes on model performance, thereby gaining a deeper understanding of the internal working mechanisms of the model. We added DSAF, the PFFM, and the SAM to the original DBNet algorithm, and then compared the model performance before the addition to evaluate the impact of these modules on the model performance.

In this paper, we conducted a series of ablation experiments on the ICDAR 2015, Total-Text, and MSRA-TD500 datasets. Performing ablation experiments on different datasets helps better validate the generalization ability of the model, facilitating a comprehensive assessment of its performance, robustness, and application scope.

For the backbone of the ablation experiments, we chose ResNet-18. Compared to ResNet-50, ResNet-18 has fewer parameters, requiring fewer computational resources for training and evaluation, resulting in faster training and inference processes. Additionally, ResNet-18 has a lower model complexity making it easier to observe the impact of each module on model performance, facilitating analysis of the ablation experiments. The results of the ablation experiments on the three datasets are presented in Table 1, Table 2 and Table 3. Since the SAM is added to the concatenated feature graph, we combined it with the PFFM in the fourth-row ablation experiment of each dataset to analyze the SAM’s impact on the overall performance of the network.

As can be seen from Table 1, on the ICDAR2015 dataset, after adding the DSAF module, the accuracy, recall, and F-score are 0.4%, 0.52%, and 0.47% higher than the original DBNet model, respectively. After employing the PFFM, the accuracy, recall, and F-score are 1.13%, 0.51%, and 0.79% higher than the DBNet model using FPN, respectively. Adding the SAM after the PFFM can further increase the accuracy, recall, and F-score to 87.53%, 78.86%, and 82.97%, respectively. By incorporating all these modules, the method used in this paper achieves an accuracy of 87.65%, a recall of 79.45%, and an F-score of 83.34% on this dataset. Compared to the original DBNet model, the addition of these modules leads to improvements in accuracy, recall, and F-score of 1.53%, 1.64%, and 1.56%, respectively.

As evident from Table 2, on the Total-Text dataset, the introduction of the DSAF module resulted in an increase of 1.44%, 2.84%, and 2.25% in accuracy, recall, and F-score, respectively, compared to the original DBNet network. After utilizing the PFFM, the accuracy, recall, and F-score were 0.62%, 3.21%, and 2.08% higher than the original DBNet model, respectively. Incorporating the SAM after the PFFM further elevated the accuracy, recall, and F-score to 87.35%, 79.28%, and 83.11%, respectively. By integrating all these modules, the method employed in this paper achieved an accuracy of 87.41%, a recall of 79.32%, and an F-score of 83.16% on this dataset. Compared to the original DBNet model, the simultaneous addition of these modules led to improvements in accuracy, recall, and F-score of 0.83%, 4.0%, and 2.61%, respectively.

As shown in Table 3, on the MSRA-TD500 dataset, the introduction of the DSAF module resulted in an increase of 0.77%, 1.57%, and 1.2% in accuracy, recall, and F-score, respectively, compared to the original DBNet network. After utilizing the PFFM, the accuracy, recall, and F-score were, respectively, 1.33%, 1.15%, and 1.23% higher than the results obtained from the DBNet model with FPN. Incorporating the SAM after the PFFM further elevated the accuracy, recall, and F-score to 87.26%, 81.58%, and 84.32%, respectively. By integrating all these modules, the method employed in this paper achieved an accuracy of 87.53%, a recall of 82.52%, and an F-score of 84.95% on this dataset. Compared to the original DBNet model, the simultaneous addition of these two modules led to improvements in accuracy, recall, and F-score of 1.78%, 2.26%, and 2.04%, respectively.

As can be seen from the three tables above, embedding the DSAF module into the feature extraction network ResNet allows us to combine local and global attention, thereby enhancing the ability to extract text feature information and better capture contextual information. Compared to the original feature fusion, the PFFM we adopted has stronger feature fusion capabilities and can significantly improve model robustness. Additionally, the SAM applied after the cascaded feature map further enhances the detection performance for diverse texts.

### 4.5. Experimental Results

In this paper, we plotted the training loss curve on three different datasets. For the backbone, we chose ResNet50. Compared to ResNet18, ResNet50 has more layers and larger parameter sizes, which enables it to have stronger feature learning capabilities and better generalization performance. Therefore, the loss curve exhibited by ResNet50 is more stable compared to that of ResNet18.

**Figure 9:**
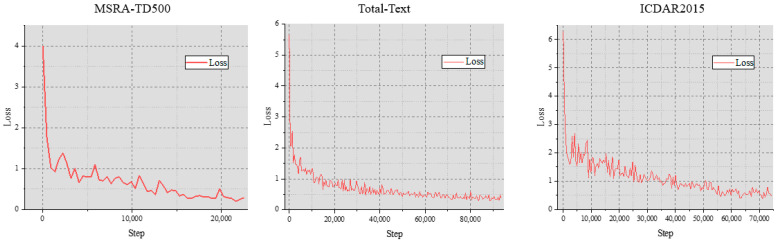
Visualization results in different types.

As can be seen from Figure 9, due to the differences in datasets and sample diversity, the convergence speed and stability of each data set are different. However, in the three datasets, the loss curve of our proposed model rapidly decreases during the initial training stage and then gradually stabilizes, indicating that the model gradually learns the data’s regularity during the training process and gradually converges to the optimal solution indirectly indicating that our proposed model has good learning ability. In addition, the loss curves trained on these three data sets can gradually reach stable convergence during the training process without large fluctuations in the later stages, which also indicates that our proposed model has good stability.

We compared the method we adopted with other methods on the multi-directional text dataset ICDAR2015, the curved text dataset Total-Text, and the multi-language text dataset MSRA-TD500. The experimental results are presented in Table 4, Table 5 and Table 6.

The comparison results of our model with other models on the multi-directional text ICDAR 2015 dataset are shown in Table 4. From Table 4, we can see that the accuracy, recall, and F-score of Ours-ResNet-18 are 87.7%, 79.5%, and 83.4%, respectively, which are 1.6%, 1.7%, and 1.7% higher than the original DB-ResNet-18. The accuracy, recall, and F-score of Ours-ResNet-50 are 87.9%, 83.4%, and 85.6%, respectively, which are 0.5%, 2.0%, and 1.3% higher than the original DB-ResNet-50. The accuracy, recall, and F-score of Ours-ResNet-50(1152) are 89.6%, 84.2%, and 86.8%, respectively, which are 1.1%, 0.4%, and 0.7% higher than the original DB-ResNet-50(1152). The experimental results show that our proposed model has better detection performance than the original model in multi-directional text detection. Compared with previous classical methods, the proposed method has achieved good results in the three indexes.

The comparison results of our proposed model with other models on the curved text Total-Text dataset are shown in Table 5. The accuracy, recall, and F-score of Ours-ResNet-18 are 87.4%, 79.3%, and 83.2%, respectively, which are 0.7%, 1.8%, and 1.7% higher than the original DB-ResNet-18. The accuracy, recall, and F-score of Ours-ResNet-50 are 88.5%, 82.4%, and 85.3%, respectively, which are 2.3%, 2.2%, and 2.1% higher than the original DB-ResNet-50. The experimental results show that our proposed model has better detection accuracy on curved text than the original model. Compared with previous classical methods, the proposed method has achieved good results in the three indexes.

The comparison results of our model with other models on the MSRA-TD500 multilingual text dataset are shown in Table 6. In Table 6, the accuracy, recall, and F-score of Ours-ResNet-18 are 87.5%, 82.5%, and 84.9%, respectively, which are 1.8%, 2.3%, and 2.0% higher than the original DB-ResNet-18. The accuracy, recall, and F-score of Ours-ResNet-50 are 89.3%, 85.2%, and 87.2%, respectively, which are 0.5%, 2.8%, and 1.7% higher than the original DB-ResNet-50. The experimental results show that our proposed model has better detection performance on multilingual text datasets. Compared with previous classical methods, the proposed method has achieved good results in the three indexes.

**Figure 10:**
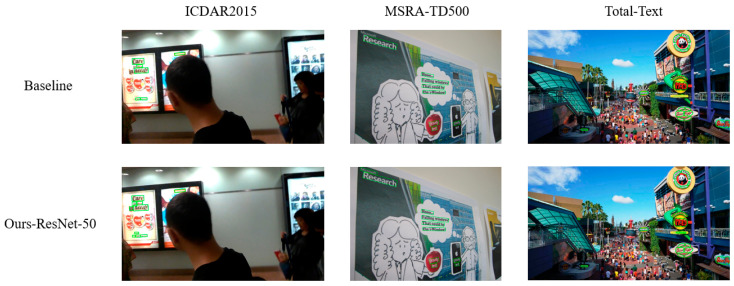
Comparison of detection results between baseline and Ours-ResNet-50.

Figure 10 below illustrates the detection results of the original DBNet model and our improved model. The figure compares the results on the ICDAR2015 dataset, the MSRA-TD500 dataset, and the Total-Text dataset, respectively. As can be seen from the comparisons, the original DBNet model exhibits missed detections in all three datasets, whereas our proposed model can better avoid such missed detections. These three images are randomly selected from the three datasets, further demonstrating the generalization ability and robustness of our model. The results show that the DSAF module, PFFM, and SAM can effectively enhance the detection ability of text features.

The above results show that the model we proposed has superior detection performance in the multi-directional text data set ICDAR2015, the curved text data set Total-Text, and the multi-language text data set MSRA-TD500. These data sets contain text information from most indoor and outdoor scenes, indicating that our model has excellent detection performance in natural scene text detection. Experiments demonstrate that the attention fusion (DSAF) module and cascade feature fusion (PFFM) module are very important for text feature extraction and feature fusion, significantly improving the detection accuracy of the original algorithm. At the same time, the added SAM also improves the detection performance to some extent. In summary, the model is superior to existing methods in performing scene text detection tasks, with superior performance, and can effectively and accurately detect text in various scenes.

## 5. Discussion and Conclusions

This paper proposes a scene text detection algorithm based on attention feature extraction and cascade feature fusion. In order to solve the problem caused by the change in text scale, we embed attention feature fusion (DSAF) in the backbone network ResNet to capture the features of different scales of text and enhance its feature extraction ability. In order to make the features more fully fused and expand the network’s receptive field, we adopt an improved cascade feature fusion (PFFM) method to solve this problem. Then, we introduce the SAM to realize spatial information interaction of different scale features. Integrating these modules into the text detection network can make the network model extract features more efficiently and improve the detection accuracy of text. The proposed method exhibits excellent performance and wide universality in publicly available scene text datasets, effectively addressing various text challenges, including long texts, curved texts, and multi-language texts. Comparative experiments show that the method has superior performance and comparable performance with advanced other methods on three datasets. The ablation experiment results also demonstrate the effectiveness of the added modules and the superiority of the improved model structure. 

In future work, we will further optimize the structure of the segmentation network, study better network models, reduce model complexity, shorten training time, reduce model parameters, and enable deployment on lightweight devices.

## Figures and Tables

**Table 1:** ICDAR2015 ablation Results.

Backbone	DSAF	PFFM	SAM	P(%)	R(%)	F(%)
Resnet-18				86.12	77.81	81.75
Resnet-18	√			86.52	78.33	82.22
Resnet-18		√		87.25	78.32	82.54
Resnet-18		√	√	87.53	78.86	82.97
Resnet-18	√	√	√	87.65	79.45	83.34

**Table 2:** Total-Text ablation Results.

Backbone	DSAF	PFFM	SAM	P(%)	R(%)	F(%)
Resnet-18				86.58	75.32	80.55
Resnet-18	√			88.02	78.16	82.80
Resnet-18		√		87.20	78.53	82.63
Resnet-18		√	√	87.35	79.28	83.11
Resnet-18	√	√	√	87.41	79.32	83.16

**Table 3:** MSRA-TD500 ablation Results.

Backbone	DSAF	PFFM	SAM	P(%)	R(%)	F(%)
Resnet-18				85.75	80.26	82. 91
Resnet-18	√			86.52	81.83	84.11
Resnet-18		√		87.08	81.41	84.14
Resnet-18		√	√	87.26	81.58	84.32
Resnet-18	√	√	√	87.53	82.52	84.95

**Table 4:** Test results for the ICDAR 2015 dataset (the values in parentheses indicate the height of the input image).

Method	P(%)	R(%)	F(%)
CTPN (Tian et al., 2016 [13])	74.2	51.6	60.9
EAST (Zhou et al., 2017 [19])	83.6	73.9	78.2
SSTD (He et al., 2017)	80.2	73.9	76.9
Corner (Lyu et al., 2018)	94.1	70.7	80.7
RRD (Liao et al., 2018)	85.6	79.0	82.2
PSE-1s (Wang et al., 2019)	86.9	84.5	85.7
PAN (Wang et al., 2019 [23])	84.0	81.9	82.9
LOMO (Zhang et al., 2019)	91.3	83.5	87.2
CRAFT (Baek et al., 2019 [26])	89.8	84.3	86.9
SAE (Tian et al., 2019)	88.3	85.0	86.6
SPCNET (Xie et al., 2019 [27])	88.7	85.8	87.2
SRPN (He et al., 2020)	92.0	79.7	85.4
FDTA (Cao et al., 2020)	81.2	89.0	84.9
FCENet (Zhu et al., 2021)	90.1	82.6	86.2
STKM (Wan et al., 2021)	88.7	84.8	86.7
DB-ResNet-18(736)	86.1	77.8	81.7
Ours-ResNet-18(736)	87.7	79.5	83.4
DB-ResNet-50(736)	87.4	81.4	84.3
Ours-ResNet-50(736)	87.9	83.4	85.6
DB-ResNet-50(1152)	88.5	83.8	86.1
Ours-ResNet-50(1152)	89.6	84.2	86.8

**Table 5:** Test results for the Total-Text dataset (the values in parentheses indicate the height of the input image).

Method	P(%)	R(%)	F(%)
TextSnake (Long et al., 2018 [21])	82.7	74.5	78.4
MTS (Lyu et al., 2018)	82.5	75.6	78.6
ATRR (Wang et al., 2019)	80.9	76.2	78.5
TextField (Xu et al., 2019)	81.2	79.9	80.6
PAN (Wang et al., 2019 [23])	89.3	81.0	85.0
CRAFT (Baek et al., 2019 [26])	87.6	79.9	83.6
CSE (Liu et al., 2019)	81.4	79.1	80.2
PSE-1s (Wang et al., 2019)	84.0	78.0	80.9
STKM (Wan et al., 2021)	86.3	78.3	82.2
DB-ResNet-18(800)	86.7	77.5	81.5
Ours-ResNet-18(800)	87.4	79.3	83.2
DB-ResNet-50(800)	86.2	80.2	83.1
Ours-ResNet-50(800)	88.5	82.4	85.3

**Table 6:** Test results for the MSRA-TD500 dataset (the values in parentheses indicate the height of the input image).

Method	P(%)	R(%)	F(%)
SegLink (Shi et al., 2017 [14])	86	70.0	77.0
DeepReg (He et al., 2017)	77.0	70.0	74.0
EAST (Zhou et al., 2017 [19])	87.28	67.43	76.08
RRPN (Ma et al., 2018)	82.0	68.0	74.0
RRD (Liao et al., 2018)	87.0	73.0	79.0
MCN (Liu et al., 2018)	88.0	79.0	83.0
PixelLink (Deng et al., 2018)	83.0	73.2	77.8
Corner (Lyu et al., 2018)	87.6	76.2	81.5
TextSnake (Long et al., 2018 [21])	83.2	73.9	78.3
PAN (Wang et al.,2019 [23])	84.4	83.8	84.1
CRAFT (Baek et al., 2019 [26])	88.2	78.2	82.9
SAE (Tian et al., 2019)	84.2	81.7	82.9
SRPN (He et al., 2020)	84.9	77.0	80.7
DRRG (Zhang et al., 2020)	88.1	82.3	85.1
FDTA (Cao et al., 2020)	71.2	84.2	77.2
MOST (He et al., 2021)	90.4	82.7	86.4
STKM (Wan et al., 2021)	81.6	77.1	79.3
DB-ResNet-18(736)	85.7	80.2	82.9
Ours-ResNet-18(736)	87.5	82.5	84.9
DB-ResNet-50(736)	88.8	82.4	85.5
Ours-ResNet-50(736)	89.3	85.2	87.2

## Data Availability

The public dataset in the experiment is available at the following connection: Total-Text: https://github.com/cs-chan/Total-Text-Dataset (accessed on 20 November 2023). ICDAR2015: https://rrc.cvc.uab.es/?ch=4&com=downloads (accessed on 8 December 2023). MSRA-TD500: http://www.iapr-tc11.org/mediawiki/index.php/MSRA_Text_Detection_500_Database_(MSRA-TD500) (accessed on 10 December 2023).

## References

[1] Zhu Y., Chen J., Liang L., Kuang Z., Jin L., Zhang W. Fourier contour embedding for arbitrary-shaped text detection. Proceedings of the IEEE/CVF Conference on Computer Vision and Pattern Recognition.

[2] Dai P., Zhang S., Zhang H., Cao X. Progressive contour regression for arbitrary-shape scene text detection. Proceedings of the IEEE/CVF Conference on Computer Vision and Pattern Recognition.

[3] Taye M.M. (2023). Theoretical understanding of convolutional neural network: Concepts, architectures, applications, future directions. Computation.

[4] Krichen M. (2023). Convolutional neural networks: A survey. Computers.

[5] Su Y., Shao Z., Zhou Y., Meng F., Zhu H., Liu B., Yao R. (2022). Textdct: Arbitrary-shaped text detection via discrete cosine transform mask. IEEE Trans. Multimed..

[6] Liao M., Wan Z., Yao C., Chen K., Bai X. Real-time scene text detection with differentiable binarization. Proceedings of the AAAI Conference on Artificial Intelligence.

[7] Karatzas D., Gomez-Bigorda L., Nicolaou A., Ghosh S., Bagdanov A., Iwamura M., Matas J., Neumann L., Chandrasekhar V.R., Lu S. ICDAR 2015 competition on robust reading. Proceedings of the 2015 13th International Conference on Document Analysis and Recognition (ICDAR).

[8] Yao C., Bai X., Liu W., Ma Y., Tu Z. Detecting texts of arbitrary orientations in natural images. Proceedings of the 2012 IEEE Conference on Computer Vision and Pattern Recognition.

[9] Ch’ng C.K., Chan C.S. Total-text: A comprehensive dataset for scene text detection and recognition. Proceedings of the 2017 14th IAPR International Conference on Document Analysis and Recognition (ICDAR).

[10] Liu W., Anguelov D., Erhan D., Szegedy C., Reed S., Fu C.-Y., Berg A.C. Ssd: Single shot multibox detector. Proceedings of the Computer Vision–ECCV 2016: 14th European Conference.

[11] Girshick R. Fast r-cnn. Proceedings of the IEEE International Conference on Computer Vision.

[12] He K., Gkioxari G., Dollár P., Girshick R. Mask r-cnn. Proceedings of the IEEE International Conference on Computer Vision.

[13] Tian Z., Huang W., He T., He P., Qiao Y. Detecting text in natural image with connectionist text proposal network. Proceedings of the Computer Vision–ECCV 2016: 14th European Conference.

[14] Shi B., Bai X., Belongie S. Detecting oriented text in natural images by linking segments. Proceedings of the IEEE Conference on Computer Vision and Pattern Recognition.

[15] Liao M., Shi B., Bai X., Wang X., Liu W. Textboxes: A fast text detector with a single deep neural network. Proceedings of the AAAI Conference on Artificial Intelligence.

[16] Liao M., Shi B., Bai X. (2018). Textboxes++: A single-shot oriented scene text detector. IEEE Trans. Image Process..

[17] Ma J., Shao W., Ye H., Wang L., Wang H., Zheng Y., Xue X. (2018). Arbitrary-oriented scene text detection via rotation proposals. IEEE Trans. Multimed..

[18] Jiang Y., Zhu X., Wang X., Yang S., Li W., Wang H., Fu P., Luo Z. R^2^ cnn: Rotational region cnn for arbitrarily-oriented scene text detection. Proceedings of the 2018 24th International Conference on Pattern Recognition (ICPR).

[19] Zhou X., Yao C., Wen H., Wang Y., Zhou S., He W., Liang J. East: An efficient and accurate scene text detector. Proceedings of the IEEE Conference on Computer Vision and Pattern Recognition.

[20] Li X., Wang W., Hou W., Liu R.-Z., Lu T., Yang J. (2018). Shape robust text detection with progressive scale expansion network. arXiv.

[21] Long S., Ruan J., Zhang W., He X., Wu W., Yao C. Textsnake: A flexible representation for detecting text of arbitrary shapes. Proceedings of the European Conference on Computer Vision (ECCV).

[22] Deng D., Liu H., Li X., Cai D. Pixellink: Detecting scene text via instance segmentation. Proceedings of the AAAI Conference on Artificial Intelligence.

[23] Wang W., Xie E., Song X., Zang Y., Wang W., Lu T., Yu G., Shen C. Efficient and accurate arbitrary-shaped text detection with pixel aggregation network. Proceedings of the IEEE/CVF International Conference on Computer Vision.

[24] Xu Y., Wang Y., Zhou W., Wang Y., Yang Z., Bai X. (2019). Textfield: Learning a deep direction field for irregular scene text detection. IEEE Trans. Image Process..

[25] Huang Z., Zhong Z., Sun L., Huo Q. Mask R-CNN with pyramid attention network for scene text detection. Proceedings of the 2019 IEEE Winter Conference on Applications of Computer Vision (WACV).

[26] Baek Y., Lee B., Han D., Yun S., Lee H. Character region awareness for text detection. Proceedings of the IEEE/CVF Conference on Computer Vision and Pattern Recognition.

[27] Xie E., Zang Y., Shao S., Yu G., Yao C., Li G. Scene text detection with supervised pyramid context network. Proceedings of the AAAI Conference on Artificial Intelligence.

[28] Long S., Qin S., Panteleev D., Bissacco A., Fujii Y., Raptis M. Towards end-to-end unified scene text detection and layout analysis. Proceedings of the IEEE/CVF Conference on Computer Vision and Pattern Recognition.

[29] Saini R., Jha N.K., Das B., Mittal S., Mohan C.K. Ulsam: Ultra-lightweight subspace attention module for compact convolutional neural networks. Proceedings of the IEEE/CVF Winter Conference on Applications of Computer Vision.

[30] Lu S., Liu M., Yin L., Yin Z., Liu X., Zheng W. (2023). The multi-modal fusion in visual question answering: A review of attention mechanisms. PeerJ Comput. Sci..

[31] Guo M.-H., Lu C.-Z., Liu Z.-N., Cheng M.-M., Hu S.-M. (2023). Visual attention network. Comput. Vis. Med..

[32] Dai Y., Gieseke F., Oehmcke S., Wu Y., Barnard K. Attentional feature fusion. Proceedings of the IEEE/CVF winter Conference on Applications of Computer Vision.

[33] Hassan E., L. L.V. (2022). Scene text detection using attention with depthwise separable convolutions. Appl. Sci..

[34] Wang F., Jiang M., Qian C., Yang S., Li C., Zhang H., Wang X., Tang X. Residual attention network for image classification. Proceedings of the IEEE Conference on Computer Vision and Pattern Recognition.

[35] Ibrayim M., Li Y., Hamdulla A. (2022). Scene text detection based on two-branch feature extraction. Sensors.

[36] Hu J., Shen L., Sun G. Squeeze-and-excitation networks. Proceedings of the IEEE Conference on Computer Vision and Pattern Recognition.

[37] Zhao H., Shi J., Qi X., Wang X., Jia J. Pyramid scene parsing network. Proceedings of the IEEE Conference on Computer Vision and Pattern Recognition.

[38] Wang Q., Ma Y., Zhao K., Tian Y. (2020). A comprehensive survey of loss functions in machine learning. Ann. Data Sci..

[39] Ho Y., Wookey S. (2019). The real-world-weight cross-entropy loss function: Modeling the costs of mislabeling. IEEE Access.

[40] Shrivastava A., Gupta A., Girshick R. Training region-based object detectors with online hard example mining. Proceedings of the IEEE Conference on Computer Vision and Pattern Recognition.

[41] Gupta A., Vedaldi A., Zisserman A. Synthetic data for text localisation in natural images. Proceedings of the IEEE Conference on Computer Vision and Pattern Recognition.

[42] Kingma D.P., Ba J. (2014). Adam: A method for stochastic optimization. arXiv.

